# Mental Health Specialist Video Consultations Versus Treatment-as-Usual for Patients With Depression or Anxiety Disorders in Primary Care: Randomized Controlled Feasibility Trial

**DOI:** 10.2196/22569

**Published:** 2021-03-12

**Authors:** Justus Tönnies, Mechthild Hartmann, Michel Wensing, Joachim Szecsenyi, Frank Peters-Klimm, Regina Brinster, Dorothea Weber, Markus Vomhof, Andrea Icks, Hans-Christoph Friederich, Markus W Haun

**Affiliations:** 1 Department of General Internal Medicine and Psychosomatics Heidelberg University Heidelberg Germany; 2 Department of General Practice and Health Services Research Heidelberg University Heidelberg Germany; 3 Institute of Medical Biometry and Informatics Heidelberg University Heidelberg Germany; 4 Institute for Health Services Research and Health Economics Centre for Health and Society Heinrich-Heine-University, Düsseldorf Düsseldorf Germany

**Keywords:** primary care, integrated care, telepsychiatry, videoconferencing, depression, anxiety, recovery, randomized controlled trial

## Abstract

**Background:**

Most people affected by depression or anxiety disorders are treated solely by their primary care physician. Access to specialized mental health care is impeded by patients’ comorbidity and immobility in aging societies and long waiting times at the providers’ end. Video-based integrated care models may leverage limited resources more efficiently and provide timely specialized care in primary care settings.

**Objective:**

The study aims to evaluate the feasibility of mental health specialist video consultations with primary care patients with depression or anxiety disorders.

**Methods:**

Participants were recruited by their primary care physicians during regular practice visits. Patients who had experienced at least moderate symptoms of depression and/or anxiety disorders were considered eligible for the study. Patients were randomized into 2 groups receiving either treatment-as-usual as provided by their general practitioner or up to 5 video consultations conducted by a mental health specialist. Video consultations focused on systematic diagnosis and proactive monitoring using validated clinical rating scales, the establishment of an effective working alliance, and a stepped-care algorithm within integrated care adjusting treatments based on clinical outcomes. Feasibility outcomes were recruitment, rate of loss to follow-up, acceptability of treatment, and attendance at sessions. Effectiveness outcomes included depression (Patient Health Questionnaire-9), anxiety (Generalized Anxiety Disorder-7), burden of specific somatic complaints (Somatic Symptom Disorder-B Criteria Scale-12), recovery (Recovery Assessment Scale-German [RAS-G]), and perception of chronic illness care (Patient Assessment of Chronic Illness Care), which were measured at baseline and 16 weeks postallocation by assessors blinded to the group allocation.

**Results:**

A total of 50 patients with depression and/or anxiety disorders were randomized, 23 in the intervention group and 27 in the treatment-as-usual group. The recruitment yield (number randomized per number screened) and the consent rate (number randomized per number eligible) were 69% (50/73) and 86% (50/58), respectively. Regarding acceptability, 87% (20/23) of the participants in the intervention group completed the intervention. Of the 108 planned video consultations, 102 (94.4%) were delivered. Follow-up rates were 96% (22/23) and 85% (23/27) for the intervention and control groups, respectively. The change from baseline scores at postmeasurement for the No Domination by Symptoms domain of recovery (RAS-G) was somewhat higher in the intervention group than in the control group (Mann-Whitney *U* test: rank-biserial *r*=0.19; 95% CI −0.09 to 0.46; *P*=.18). We did not detect any notable differences between the intervention and control groups in terms of other effectiveness outcomes. We did not observe any serious adverse events related to the trial.

**Conclusions:**

The intervention and study procedures were found to be feasible for patients, primary care practice staff, and mental health specialists. A sufficiently powered pragmatic trial on mental health specialist video consultations should be conducted to investigate their effectiveness in routine care.

**Trial Registration:**

German Clinical Trials Register DRKS00015812; https://www.drks.de/drks_web/navigate.do?navigationId=trial.HTML&TRIAL_ID=DRKS00015812.

## Introduction

### Primary Care Mental Health

Depression and anxiety disorders are two of the three most prevalent mental disorders and cause substantial global and individual disease burden [1]. Patients with depression or anxiety disorders are often treated exclusively in primary care, which brings the primary care physician in a crucial position for mental health care [2-4]. Most primary care physicians provide comprehensive care to their patients. However, a substantial proportion of patients with severe conditions and somatic comorbidities are not adequately treated. They need more specialized care; however, their access is often impeded by (1) long waiting times at the provider’s end, (2) older patients’ immobility because of increasing multimorbidity in an aging society, and (3) an emphasis on assessment and treatment of somatic symptoms because of guideline recommendations [5,6]. To resolve these challenges, it is essential to develop health care models that combine the easily accessible environment of primary care and the expertise in timely diagnostics and therapy of a mental health specialist.

Health care models, which may provide a tailored treatment for patients initially presenting to their primary care physician, have been developed. In some of these models (eg, collaborative care), the primary care physician is supported by a care manager, who tracks patients per telephone, conducts psychological assessments, and presents the data to a mental health specialist, often a psychiatrist [7,8]. The mental health specialist monitors the patients by scanning the case reports and can intervene, if necessary, by prescribing drugs or scheduling face-to-face consultations. In other models, the primary care team and mental health specialist are colocated [9-11]. The mental health specialist provides team-based specialized treatment as a routine part of primary care, such as goal setting together with patients, patient activation, and psychosocial care. In the practice, direct cooperation allows patients to be referred by warm handoffs instead of conventional referral forms. Regardless of whether a mental health specialist is locally present, these health care models provide more direct access to specialized care for mental health patients and foster cooperation between primary and specialized mental health care.

These integrated care models are promising and have been successfully implemented, particularly in the US health care system. However, small and remote primary care practices struggle with the implementation of these care models. In European countries, such as the United Kingdom, France, and Germany, where the mean number of physicians per practice is lower than that in the United States, practices with 1 or 2 doctors often do not have the financial resources to employ an additional mental health specialist [12]. Especially in the German health care system, integrated mental health care models have rarely been implemented so far. Therefore, it is essential to develop and evaluate innovative modes to put these integrated care approaches into practice.

### Mental Health Specialist Video Consultations

Real-time video consultations conducted by mental health specialists have been shown to be a promising approach to integrated care. This technology-supported mode of delivery is increasingly considered as an alternative to face-to-face settings. We conducted a thorough literature review and identified 315 records. A total of 11 records were relevant, and among these, 6 systematic reviews show that, in general, telemedicine interventions for mental health conditions seem to be effective [13-18]. Concerning the integration of telepsychiatry services in primary care, several observational and interventional studies have demonstrated that mental health specialist video consultations contribute to overcoming geographical barriers and treating the increasing number of multimorbid patients often cut off from specialized care [19-24]. Randomized trials evaluating video consultations have been conducted either in the unique setting of the US Veterans Health Care Administration in Rural Federally Qualified Health Centers [19,20] or included patients from inpatient health care settings [23,24]. The implementation of telemedical approaches within mental health care has generally been promoted more in the United States than in Europe through passing guidelines by the American Telemedicine Association [25]. In particular, for European primary health care settings, the results of those settings can therefore only be generalized to a limited extent.

### Purpose of the Study

Consequently, the aim of this study is to evaluate if and how mental health specialist video consultations and primary care can be integrated into a European health care system. Therefore, we conducted a randomized controlled feasibility trial in Germany by implementing mental health specialist video consultations in 5 primary care practices. If the intervention proves to be feasible, the results of this trial will inform the planning and setup of a subsequent larger randomized controlled prospective trial to evaluate efficacy.

## Methods

### Trial Design and Participants

We conducted an assessor-blinded, randomized, prospective, parallel group feasibility PROVIDE-B (improving cross-sectoral collaboration between primary and psychosocial care: an implementation study on video consultations-B) trial between March 1 and October 7, 2019, in 5 primary care practices in the State of Baden-Wuerttemberg in Southern Germany [26]. Primary care physicians were either recruited during a preceding qualitative preimplementation study [27] or through a network of collaborating academic research practices affiliated with the Department of General Practice and Health Services Research at Heidelberg University. We sent an invitation letter and visited interested practices to inform the practice teams about the study, including the concomitant process evaluation and the assessments involved. We also tested the quality of the internet connection to evaluate eligibility. We recruited 4 mental health specialists at the Institute for Psychotherapy, Heidelberg, which is a state-approved psychotherapeutic training facility at Heidelberg University. Mental health specialists were clinical psychologists with a diploma or master’s degree in psychotherapy training or resident doctor training for board certification in psychosomatic medicine and psychotherapy, which is an independent specialty in Germany. All participating specialists had at least 2 years of training. Although specialists were not allowed to prescribe medication because of regulatory reasons, they had the possibility to suggest starting the patient on medication or changing their medication.

Eligible patients (1) exceeded cutoffs of 9 points for the Patient Health Questionnaire-9 (PHQ-9) and/or for the Generalized Anxiety Disorder-7 (GAD-7), respectively [28], which represents at least moderately severe symptom burden by either disorder; (2) did not yet have mental health treatment or, until the date of commencement of the study, insufficient treatment (psychotherapy, psychopharmacotherapy, or both) or difficulty with adherence to treatment; (3) agreed to participate in the study by written informed consent; (4) were capable of giving consent; and (5) were aged 18 years or older. Exclusion criteria for patients were (1) substance abuse/dependence that is likely to compromise intervention adherence; (2) risk of endangerment to others and/or risk of self-endangerment; (3) need for emergency medical treatment, for example, admission; (4) acute psychotic symptoms, for example, persecutory delusions and/or thought insertion; (5) severe cognitive impairment or dementia; (6) significant hearing and/or visual impairment; (7) pregnancy in the second trimester or later; and (8) insufficient German language proficiency. To ensure maximum generalizability, general practitioners as experts for their patients decided whether treatment was insufficient or whether there were difficulties with adherence. All other inclusion and exclusion criteria were assessed through standardized computer-assisted telephone interviews conducted by a study team member. The PROVIDE-B trial protocol was approved by the Medical Faculty of the University of Heidelberg Ethics Committee (S-634/2018) and was subsequently published [26].

### Randomization and Masking

The participants were recruited via their primary care physicians during regular visits in the practice. On the basis of their clinical judgment, GPs prospectively selected individuals suspected to be affected by depression or anxiety and presented the study to them by offering information material. After providing written informed consent, eligible participants were randomly assigned (1:1) to the video consultation model versus treatment-as-usual via a secure, web-based randomization system (Randomizer V.2.0.2) operated by a data manager at the Institute of Medical Biometry and Informatics, Heidelberg University. We used block randomization stratified by primary care practice, with a block size of 4. Randomization at the individual level was independent and concealed. Allocation was subsequently made known to the principal investigator (M Haun), trial coordinator (JT), and mental health specialists. Participants, mental health specialists, and primary care practice staff were informed of the allocation by phone or email. Telephone interviews were used to assess the baseline data before randomization. Two research assistants, masked to group allocation, conducted the postmeasurement in telephone interviews with the participants.

### Procedures

Participants allocated to the mental health specialist video consultations were offered up to 5 sessions with a mental health specialist during a 3-month treatment window. If patients and mental health specialists agreed that no further treatment was required, they were allowed to end the consultations as early as after the third session. The intervention featured web-based, real-time video consultations involving a 2-way interactive video to a primary care practice between mental health specialists and patients. Apart from that, the intervention was fairly similar to conventional consultation-liaison models in mental health primary care [29] and the collaborative care model [30,31], which both constitute a trade-off between increasing involvement of the primary care clinician on the one hand and increasing involvement of the mental health specialist on the other hand [32]. Both models, such as the PROVIDE-B intervention, target well-defined disorders that are associated with some degree of disability but for which effective treatments are available. Nevertheless, in contrast to consultation-liaison and collaborative care services where mental health specialists act as advisors to primary care physicians (eg, care managers in collaborative care), our intervention included more therapeutic aspects. Specifically, the intervention included 3 core intervention elements (*active ingredients*) for effective primary care–based mental health care, namely (1) systematic diagnosis plus proactive monitoring using validated clinical rating scales, (2) the establishment of an effective working alliance, and (3) a stepped-care algorithm within integrated care adjusting treatments based on clinical outcomes. If indicated, the intervention also included brief psychological therapy that worked with interpersonal dynamics, which has been shown to confer additional benefits [8]. When the patient had a more chronic condition that demanded long-term treatment, the mental health specialists and the patients mutually developed a care plan that, if indicated, included transition to secondary specialist care. Furthermore, the mental health specialist discussed cases with primary care physicians. The intervention followed a transdiagnostic treatment approach for emotional disorders (depression and anxiety), for which various meta-analyses have shown efficacy compared with control conditions on measures of overall anxiety, disorder-specific anxiety, and depression [33,34]. In addition, the intervention entailed elements from problem-solving therapy, which has been shown to yield moderate effects in alleviating depression and anxiety in primary care [35]. Psychodynamic elements following a relationship focus and interpersonal understanding were added to foster the working alliance, which has been promoted as a crucial element of manuals achieving high acceptability in both patients and clinicians. At the end of the consultations, the mental health specialist proceeded as laid out in the care plan, providing a treatment summary and tailored recommendations to both the patient and the primary care physician. The intervention was conducted in line with the *Best Practices in Videoconferencing-Based Telemental Health* issued by the American Psychiatric Association and the American Telemedicine Association [25]. In line with the stage model of psychotherapy manual development, we compiled a stage I intervention manual delineating treatment techniques, goals, and format (the manual is available in the study by *Tönnies* et al [26]). For the description of the intervention, we followed the template for intervention description and replication guidance [26,36]. A structured description of the intervention is presented in Multimedia Appendix 1.

Patients received their first video consultation shortly after randomization and were scheduled for up to 5 sessions, lasting 50 minutes each, at biweekly intervals. The video consultations were conducted on a secure (ie, encrypted), web-based videoconferencing platform on a subscription basis (arztkonsultation ak GmbH;) at the fixed time slots set by the primary care practice staff. The patients were in a designated room in the general practice and the mental health specialists in either their office/private practice or another suitable, designated room at home. For every video consultation, patients received a transaction authentication number to log on to the encrypted, web-based videoconferencing platform for clinical video consultations. As the platform was easy to access, patients who had different levels of experience with videoconferencing had no major difficulties with logging in. Each mental health specialist was permanently assigned to one primary care practice. After the third session, we conducted an interim evaluation of the symptoms (using the PHQ-9 and GAD-7) and sent the results to the mental health specialist to tailor the treatment accordingly. After the last consultation with the patient, the mental health specialist sent a written case summary to the primary care practice, which was then attached to the medical record and on which, if needed, further decisions on follow-up procedures were based. Parallel to the study, mental health specialists received weekly group supervision led by a senior consultant in psychiatry and psychosomatic medicine from the Department of General Practice and Psychosomatics, Heidelberg University. Patients allocated to the control group were informed that they would receive the usual care provided by their primary care physicians. This might or might not have included a referral to a mental health specialist or other psychosocial treatment outside the study. The respective primary care physician was also informed about the group to which the patient was allocated. There were no restrictions on the usual treatment by primary care physicians.

### Outcomes

The main outcome was the feasibility of a mental health care model integrating mental health specialist video consultations and primary care, which we operationalized by applying early stage implementation outcomes [37]:

- Recruitment strategy and recruitment rate (efficiency of recruitment strategies).
- Intervention acceptability in patients (attendance of sessions for the intervention arm).
- Acceptability of outcome measurements (rate of loss to follow-up and feedback after assessments).
- Intervention safety in patients (Inventory for the Assessment of Negative Effects of Psychotherapy [INEP]).
- Feasibility of study procedures, including the intervention (qualitative process evaluation will be published elsewhere).

In addition to feasibility, the measurements of effectiveness were also included. Effectiveness outcomes were depressive (PHQ-9) and anxiety (GAD-7) symptom severity, burden of specific somatic complaints (Somatic Symptom Disorder-B Criteria Scale [SSD-12]) [38], and recovery (Recovery Assessment Scale [RAS-G]), defined as “the personal process of adaptation and development through which the individual overcomes the negative personal and social consequences of [a] mental disorder and regains a self-determined and meaningful life” [39] consisting of 5 subdomains (more details on the domains are given in Multimedia Appendix 2 [40]) and “the quality and patient-centeredness of chronic illness care” (Patient Assessment of Chronic Illness Care [PACIC]) [41]. Health-related quality of life was measured using the European Quality of Life 5 Dimensions [42]. This also included a visual analog scale ranging from 0 to 100, on which the patients rated their quality of life with 0 for the lowest quality and 100 for the highest quality. Intervention-related costs and health care usage, including use of service and medication prescribing, were measured using the Questionnaire for the Assessment of Medical and Nonmedical Resource Utilization in Mental Disorders [43]. All these outcomes were assessed at baseline and 16 weeks postallocation. By choosing this period, we sought to (1) ensure that the intervention was completed despite possible delays (eg, because of time-consuming appointment management, patients’ and providers’ vacations) and (2) be able to assess not only immediate but also long-term effects. For the intervention group, intervention safety (unintended consequences and adverse effects) was assessed during close-out measurement by applying INEP [44]. INEP comprises 21 items asking the participant how they assess the effects of a psychosocial intervention.

### Statistical Analysis

We based the sample size on recommendations for obtaining reliable sample size estimates in feasibility studies, which indicated that 50 patients would be needed (ie, 25 in each group) [45]. The primary analysis followed the intention-to-treat principle.

First, as part of the data preparation, we applied an available-item strategy to calculate the total scale scores [46]. In this feasibility trial, we used pairwise deletion as a missing data strategy and did not adjust for multiple testing in the analyses. Second, we computed descriptive statistics for the feasibility outcomes, summarizing results for discrete variables in absolute and relative frequencies, and for continuous variables in means, SDs, medians, and IQRs. Third, we conducted assumption checks (screening for normality and equality of variances) for all variables of effectiveness outcomes. To investigate differences in effectiveness outcomes, we compared the change between baseline assessment and postassessment of PHQ-9, GAD-7, RAS-G, SSD-12, and PACIC in both groups using Mann-Whitney *U* tests [47,48]. We applied the screening values for computing the PHQ-9 and GAD-7 change scores if the baseline assessment had been performed no later than 28 days after screening. To increase interpretability, change scores were calculated by taking the difference between baseline assessment and postassessment scores or between postassessment and baseline assessment scores, depending on the respective scale. Therefore, a positive change indicates an improvement between the baseline assessment and postassessment. For the effect size *r* (rank-biserial correlation coefficient), the following interpretation applies: if *r*>0 in the baseline or follow-up scores, the health status in the intervention group was better than that in the control group. If *r*>0 for the change score, the improvement in the intervention group was larger than that in the control group.

We used R (version 4.0.2), JASP (version 0.12.2) [49,50], and Stata (version 15.1) for all analyses. This trial was prospectively registered with the German Clinical Trials Register (registration no. DRKS00015812). We did not implement any changes to the methods after trial commencement. We have reported this trial in accordance with the CONSORT (Consolidated Standards of Reporting Trials) extension for randomized pilot and feasibility trials (see the checklist given in Multimedia Appendices 3 and 4) [51].

## Results

### Sample Description

**Figure 1:**
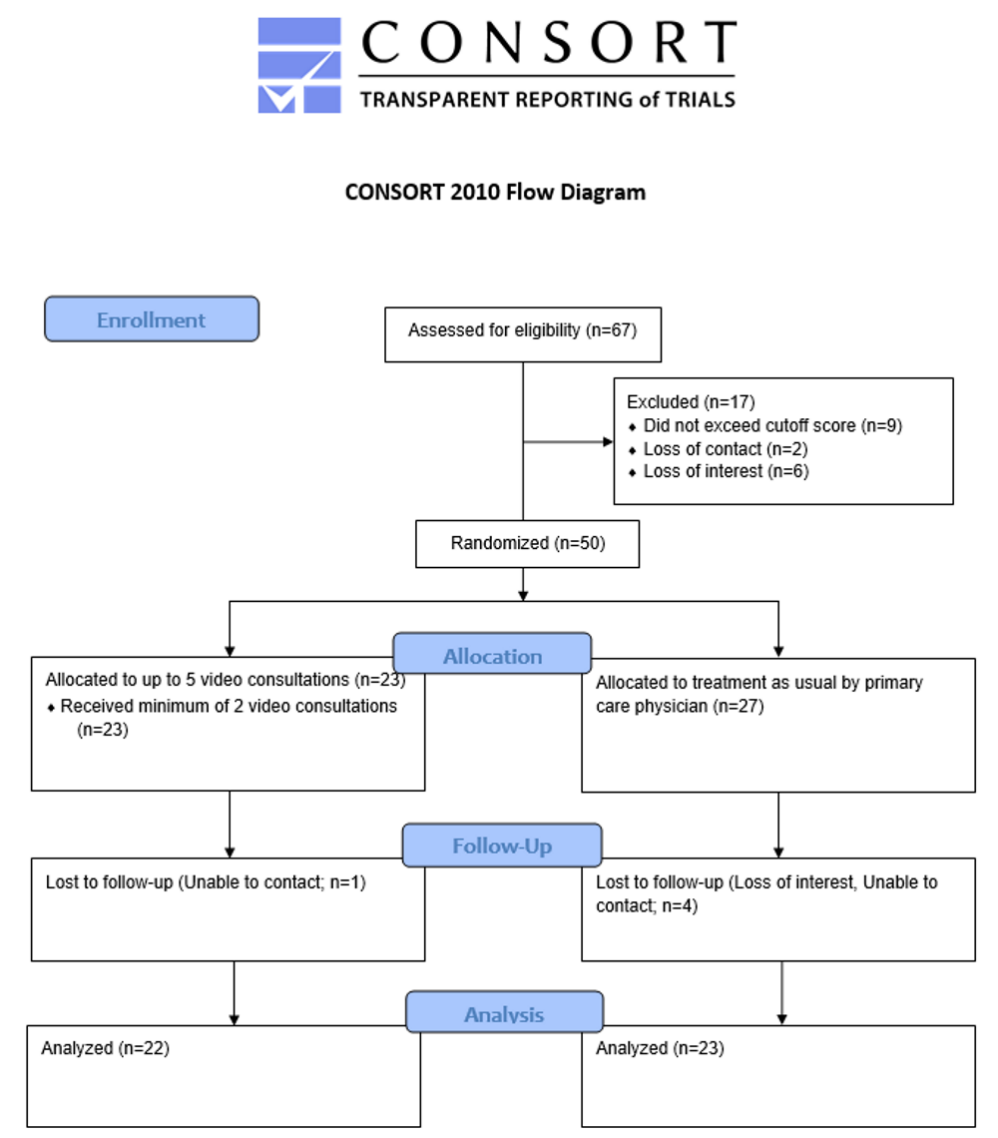
CONSORT (Consolidated Standards of Reporting Trials) flow diagram.

Of the 70 approached primary care practices, 12 were interested in participation. This relatively low rate may be explained by the fact that the provision of a designated room for a fixed time slot of 4 hours per week and a stable internet connection were mentioned as obligatory inclusion criteria. Outside the fixed time slot, the practice could use the room for routine clinical care. Some practices might not have been able to meet these requirements; therefore, they did not reply in the first place. Supporting this assumption, a preimplementation survey among primary care practitioners showed that more than half of them had no designated room available for video consultations [52]. After screening, we included 5 practices. Reasons for exclusion were a lack of designated rooms and/or internet connectivity. We recruited 50 participants—23 were randomized to mental health specialist video consultations and 27 to treatment-as-usual (Figure 1; Table 1). A total of 96% (48/50) participants had at least moderate levels of both depressive (PHQ-9≥10) and anxiety (GAD-7≥10) symptom severity, whereas 4% (2/50) participants were affected by moderate levels of depressive symptom severity only.

**Table 1:** Baseline characteristics (N=50).

Variable		Intervention group (n=23)	Control group (n=27)	Overall (N=50)
**Age (years)**				
	Mean (SD)	45.9 (15.86)	51.2 (15.46)	48.8 (15.71)
	Median (range)	48 (22-72)	56 (18-72)	54 (18-72)
**Gender, n (%)**				
	Female	16 (69.6)	19 (70.4)	35 (70)
	Male	7 (30.4)	8 (29.6)	15 (30)
**Marital status, n (%)**				
	Single	5 (21.7)	5 (18.5)	10 (20)
	In partnership	18 (78.3)	22 (81.5)	40 (80)
**Education level, n (%)**				
	9 years or less	6 (26.1)	14 (51.9)	20 (40)
	More than 9 years	15 (65.2)	13 (48.1)	28 (56)
	Missing	2 (8.7)	0 (0)	2 (4)
**Employment status, n (%)**				
	Employed	13 (56.5)	9 (33.3)	22 (44)
	On sick leave	3 (13)	5 (18.5)	8 (16)
	Retired	4 (17.4)	5 (18.5)	9 (18)
	Unemployed	2 (8.7)	2 (7.4)	4 (8)
	Missing	1 (4.3)	6 (22.2)	7 (14)
**Number of chronic diseases**				
	Mean (SD)	0.9 (1.06)	1.4 (1.33)	1.1 (1.22)
	Median (range)	1 (0-3)	1 (0-4)	1 (0-4)
	Missing	0 (0)	1 (3.7)	1 (2)
**Current psychiatric treatment or psychotherapy, n (%)**				
	No	20 (87)	21 (77.8)	41 (82)
	Yes	3 (13)	6 (22.2)	9 (18)
**Past psychiatric treatment or psychotherapy, n (%)**				
	No	7 (30.4)	9 (33.3)	16 (32)
	Yes	13 (56.5)	12 (44.4)	25 (50)
	Declined to answer	2 (8.7)	3 (11.1)	5 (10)
	Missing	1 (4.3)	3 (11.1)	4 (8)
**Current psychopharmacological treatment, n (%)**				
	No	15 (65.2)	14 (51.9)	29 (58)
	Yes	8 (34.8)	12 (44.4)	20 (40)
	Missing	0 (0)	1 (3.7)	1 (2)
**Past psychopharmacological treatment, n (%)**				
	No	9 (39.1)	12 (44.4)	21 (42)
	Yes	5 (21.7)	4 (14.8)	9 (18)
	Declined to answer	5 (21.7)	5 (18.5)	10 (20)
	Missing	4 (17.4)	6 (22.2)	10 (20)
**Willingness to accept psychotherapy, n (%)**				
	Disagree	1 (4.3)	1 (3.7)	2 (4)
	Agree	4 (17.4)	3 (11.1)	7 (14)
	Strongly agree	17 (73.9)	20 (74.1)	37 (74)
	Missing	1 (4.3)	3 (11.1)	4 (8)
**Willingness to accept psychopharmacological treatment, n (%)**				
	Strongly disagree	5 (21.7)	4 (14.8)	9 (18)
	Disagree	6 (26.1)	5 (18.5)	11 (22)
	Agree	5 (21.7)	3 (11.1)	8 (16)
	Strongly agree	6 (26.1)	9 (33.3)	15 (30)
	Missing	1 (4.3)	6 (22.2)	7 (14)
**Level of depressive symptoms (PHQ-9^a^), n (%)**				
	Blank	1 (4.3)	0 (0)	1 (2)
	Mild	1 (4.3)	4 (14.8)	5 (10)
	Moderate	17 (73.9)	10 (37)	27 (54)
	Severe	3 (13)	11 (40.7)	14 (28)
	Highly severe	1 (4.3)	2 (7.4)	3 (6)
**Level of anxiety (GAD-7^b^), n (%)**				
	Blank	2 (8.7)	2 (7.4)	4 (8)
	Mild	8 (34.8)	5 (18.5)	13 (26)
	Moderate	10 (43.5)	12 (44.4)	22 (44)
	Severe	3 (13)	8 (29.6)	11 (22)

^a^PHQ-9: Patient Health Questionnaire-9.

^b^GAD-7: Generalized Anxiety Disorder-7.

### Recruitment, Rate of Loss to Follow-Up, and Success of Blinded Assessment

The overall recruitment yield (number randomized per number screened) was 69% (50/73), the recruitment rate (number recruited and randomized per primary care practice per month) was 50/(4×7+1×5)=1.52. The consent rate (number randomized per number eligible) was 86% (50/58). We did not have to employ any additional recruitment routes in addition to direct recruitment by primary care physicians. With 1 dropout in the intervention group (1/23, 4% could not be reached/reason unknown) and 4 dropouts in the control group (4/27, 15%; reasons: 3 lost interest and 1 could not be reached/reasons unknown), follow-up rates were 95.7% and 85.2% for the intervention and control groups, respectively. The overall follow-up rate was 90%. Unintentional unblinding of the actual randomly assigned group during postmeasurement occurred in 13% (4/45) of retained cases. Of these, 5 blind breaks were in the video consultation group and 1 was in the control group. The period of recruitment and intervention was between March 1 and October 7, 2019. The last follow-up measurement was conducted on February 10, 2020.

### Acceptability of Treatment, Attendance at Sessions, and Reasons for Dropout

Retention in the video consultation group was reasonable, with 87.0% (20/23) of the participants completing the intervention as planned (regardless of availability of follow-up data). In total, 8.7% (2/23) participants attended only the first 2 sessions (1 experienced persistent connectivity failures; 1 expected long-term therapy and was dissatisfied with the length of the intervention). Of the 23 participants, 1 (4.3%) stopped after the third session for unknown reasons. Participants who were allocated to the 50-minute video consultation received an average of 4.4 sessions (SD 0.9; range 2-5 consultations). Of the 108 planned video consultations, 102 (94.4%) were successfully delivered. For completers, the median interval between the initial and final video consultation amounted to 49.5 days (range 21-70 days). In the intervention group, 35% (8/23) of the patients received some form of specialist mental health care (defined as at least one visit to a psychiatrist or psychotherapist as measured on the Questionnaire for the Assessment of Medical and Nonmedical Resource Utilization in Mental Disorders) outside the study. Of the 27 participants in the control group, 12 (44%) received some form of specialist mental health care. The patients had no major difficulties in responding to the questionnaires applied as part of the outcome measurement. They evaluated the assessments as feasible and appropriate. Considering all data for the change from baseline scores at follow-up, the highest fraction of missing information was found for the *No Domination by Symptoms* domain of recovery (RAS-G), amounting to 7% (3/45) of cases.

### Effectiveness and Health Economic Outcomes

The findings for the effectiveness outcomes and health economics are presented in Multimedia Appendices 5 and 6 [43,53], respectively. Change from baseline scores for the “No Domination by Symptoms” domain of recovery (RAS-G) were somewhat higher at postmeasurement for the video consultation group (mean change score 1.8, SD 2.56) compared with the control group (mean change score: 0.9, SD: 2.30; Mann-Whitney *U* test: rank-biserial *r*=0.19; 95% CI −0.09 to 0.46, 75% CI 0.02-0.35, *P*=.18). We did not detect any notable differences between the intervention group and the control group for the other effectiveness outcomes. Regarding the use of services outside the trial, the number of psychiatric outpatient clinic contacts seems to be larger at follow-up than at baseline for both groups. However, only 3 individuals in the intervention group had 21 contacts. The 7 contacts in the control group were induced by 2 individuals. The sum of provided specialist mental health care by psychotherapists, specialists in psychosomatic medicine, and psychiatrists is larger in the control group (baseline: 29; follow-up: 61) than in the intervention group (baseline: 7; follow-up: 38), which is again driven by few individuals (individuals of the control group with at least one specialist mental health care contact at baseline [n=6] and at follow-up [n=12]; individuals of the intervention group with at least one specialist mental health care contact at baseline [n=6] and at follow-up [n=8]).

### Unintended Consequences and Adverse Effects

Self-report data for unintended consequences and adverse effects, as measured on the INEP at 16 weeks postallocation, were available for 96% (22/23) of the participants assigned to video consultations. Considering all 21 INEP items, 18% (4/22) of these participants reported at least one unintended consequence or adverse effect attributed to the intervention instead of their life circumstances (average number of adverse effects per patient 0.3, SD 0.7). One participant reported that she or he “feels worse” at the end of the intervention and that they were depending too much on their mental health specialist. A second participant stated that they “felt hurt” by the mental health specialist’s statements and that they experienced longer periods of feeling down during or after the intervention. A third participant indicated that they feared that colleagues could find out about them being in treatment and that they experienced longer periods of feeling down during or after the intervention. A fourth participant reported being affected “from events in her/his past more than in the time before the intervention.” Of the 22 participants in the intervention group for whom data were available, 18 (81%) did not report any unintended consequences or adverse effects attributed to the intervention. Notably, we did not observe any serious adverse events (ie, sexual harassment by mental health specialists, self-endangerment, and/or endangerment to others).

## Discussion

### Principal Findings

In this assessor-blinded, randomized controlled feasibility trial, we found that a study comparing mental health specialist video consultations and treatment-as-usual by primary care physicians is feasible in people presenting with depression and/or anxiety in primary care. The feasibility of a subsequent definitive randomized controlled trial providing robust information on effectiveness is underscored by a reasonable recruitment yield, the high level of consent among eligible patients, and most importantly high levels of intervention acceptability and a low rate of loss to follow-up, which was slightly more pronounced in the control group. We attribute this to the integration of mental health specialists and primary care physicians, which accounted for seamless referrals from primary care to specialized care. Mental health specialist video consultations were generally safe and well accepted by both patients and health professionals. Although this feasibility trial was not formally powered to assess the evidence of a clinical response, the preliminary outcome data point to the benefits of being empowered to cope with symptoms. For the remaining outcomes, we did not find notable differences between the intervention and control groups, which may be explained by the fact that more patients in the control group had already been receiving psychiatric treatment, psychotherapy, and/or psychopharmacological treatment at enrollment compared with the intervention group. Even if we have found significant differences in our feasibility trial, it would have been inappropriate to interpret them as such because of the small and not formally calculated sample size in pilot or feasibility trials [54]. As our feasibility study covered several aspects of a full-scale randomized controlled trial and we present different outcomes, it is similar to a pilot trial. However, because our main objective was to test the feasibility of a mental health care model and to find aspects that may improve the implementation in the upcoming main trial, this study meets the characteristics of a feasibility trial. In addition, the fact that we conducted a parallel qualitative process evaluation indicates a feasibility trial [55].

### Limitations

This feasibility trial had several limitations. First, with respect to generalizability, we had to draw on a nonprobability sample for all participants, including practices and mental health specialists. In this regard, we cannot fully rule out volunteer bias, that is, participating stakeholders exhibiting a higher openness toward web-based delivery of care compared with the respective underlying population. However, at this stage, our main goal is to evaluate feasibility, which usually builds on the motivation and engagement of innovators who are less reluctant to depart from the conventional paradigm of face-to-face clinical encounters. Some authors have explicitly encouraged trialists to focus on innovators as opposed to losing time on so-called laggards in the pilot phase of telepsychiatry programs [56]. Second, we did not systemically observe or measure fidelity to the intervention as laid out in the intervention manual to prevent implementation failure because we regarded video and/or audio recording of the sessions as too disruptive for the therapeutic process [57,58]. Although we cannot fully rule out inadequate implementation, together with the supervisor, the principal investigator (M Haun) did assess the content of the sessions in the weekly supervision. However, in the sufficiently powered effectiveness trial, we will implement a systematic self-report fidelity assessment for mental health specialists at the end of each video consultation, enabling us to determine the extent to which the results will be because of the study intervention and to further increase statistical power [59]. Most importantly, we will monitor and foster continuous adherence to the fidelity plan throughout the trial. Third, patients’ intervention acceptability was measured by the number of sessions attended. However, patients might have attended mental health video consultations despite finding them not useful. As this was the only measure of patients’ acceptability, more detailed statements on how the patients evaluate the consultations will not be available until the results of the qualitative process evaluation are published. Fourth, it is not clear to which degree participants in the intervention group received more attention than those in the control group. A potential clinical improvement in the intervention group therefore does not necessarily have to be caused by the intervention but may be because of greater attention. However, following the recommendation for pragmatic trials in mental health services research, the definition of the control condition as treatment-as-usual was deliberately broad and was supposed to be as equal as possible to routine care [60]. Therefore, we tried not to interact with the patients at all and did not assess more information about the potential attention they might have received, for example, by using other health services during the trial. Nevertheless, in the sufficiently powered main trial, we will include health care service use in subgroup analyses with respect to attention received by control group subjects. Fifth, during the trial, some patients in both groups received other psychosocial care. As the intervention aimed to provide triage and, if indicated, facilitate the transition to specialist mental health care and the control condition was defined as treatment-as-usual, the use of treatment outside the trial was not excluded. As described, we did not collect data on the use of services in great detail and therefore did not include those in the analysis of clinical outcomes. However, we will include these data in subgroup analyses in a sufficiently powered main trial to investigate the potential clinical impact of psychosocial services use.

### Comparison With Previous Work

The findings of this trial concur with results on feasibility from previous trials and synthesized findings from reviews. A large systematic review on telehealth interventions in mental health analyzed 5 full-scale randomized controlled trials using video consultations for various mental health conditions in settings other than primary care. In all of these trials, video consultations were reported as well accepted by different populations and under different conditions [61-64]. The few trials that specifically integrated mental health specialist video consultations in primary care also yielded substantial acceptance of and satisfaction with this new form of technology-based care [19,20,65,66]. Nevertheless, all these trials were conducted in the United States and/or drew on samples from specific, in part, high-structured contexts (eg, military, including veterans). It is very likely that the patient population in primary care and the contextual factors of how primary care is organized (eg, single-practitioner models in Europe) differ in many other Western countries [12,67]. Specifically, neither collaborative care nor integrated care models are commonly used in Germany or other European health care systems. Thus, our intervention comprising the integration of specialized and primary care combined with the video-based mode of delivery can be considered relatively innovative. In this regard, the findings of our trial, that is, high retention, no major adverse effects, and high satisfaction, show that video-based integrated care models are feasible more broadly, even in health care systems with a low level of experience in integrated care. Against the background of the debate on which patient populations video consultations might be suitable for, particularly pertaining to older aged and/or severely burdened patients [68], our sample proved to be quite heterogeneous (eg, in terms of age and socioeconomic status). Overall, the findings of this feasibility trial indicate that even severely burdened patients can be reached through mental health specialist video consultations in primary care. In this regard, our intervention involved patients from difficult-to-reach populations who might have never been engaged in specialized treatment following conventional care pathways [69,70]. Indeed, 1 in 3 of the participants in our study had never sought specialized mental health treatment before enrollment in our trial. An additional strength of this feasibility trial was the innovative, systematic assessment of adverse and negative effects and harms and their potential attribution to the intervention itself using a validated self-report instrument. Although calls for such an assessment in clinical trials are continuously put forward, there is some evidence that in the field of psychotherapy, only a small proportion of studies actually report unintended consequences or adverse effects [71]. We found that 18.2% of all participants (average number of adverse effects per patient 0.3, SD 0.7) reported at least one unintended consequence or adverse effect attributed to the intervention, which is (1) much less than the prevalence of 70.5% for INEP in a clinical sample (average number of adverse effects per patient 2.1, SD 2.2) [72] and (2) well within the range of 0% to 25% reported for intervention groups in psychotherapy trials [71]. However, in digital health interventions, the impact of the patient-clinician relationship has scarcely been investigated [73]. Therefore, it is not clear whether unintended consequences or adverse effects are caused by the mode of delivery through videoconferencing or by failed rapport between the patient and clinician. At any rate, technology-supported interventions are challenging for the patient-clinician relationship, and this requires investigation regarding the negative or adverse effects of psychotherapy. Notwithstanding, interpreting unintended consequences or adverse effects remains to be a unique challenge in psychotherapy interventions, where the sound delivery of treatment may nevertheless be linked to patients reporting such effects [74].

### Conclusions

A study comparing mental health specialist video consultations and treatment-as-usual by primary care physicians in patients with depressive and anxiety disorders is feasible. The main implication of this trial is that a sufficiently powered effectiveness trial is needed to provide evidence about the relative efficacy of mental health video consultations in primary care. In our trial, the intervention proved to be unobtrusive and compatible with normal practice. Participants from various socioeconomic and cultural backgrounds could be enrolled so that a definitive trial should aim more broadly at the primary care patient population by applying pragmatic eligibility criteria. Indeed, we have embarked on a full-scale effectiveness trial in which 320 patients will be enrolled (NCT04316572), which will also include a health economic evaluation. Having applied a conservative sample size calculation, we accounted for loss to follow-up by inflating the recruitment by 20%. From a clinical perspective, at present, it seems reasonable and safe to offer video consultations to patients who cannot assess specialist services using conventional pathways.

## Appendix

Multimedia Appendix 1 Structured description of the intervention.

Multimedia Appendix 2 Structured description of the domains of the Recovery Assessment Scale, German Version.

Multimedia Appendix 3 CONSORT (Consolidated Standards of Reporting Trials) checklist, an extension for randomized pilot and feasibility trials.

Multimedia Appendix 4 CONSORT (Consolidated Standards of Reporting Trials) eHealth checklist (V.1.6.1).

Multimedia Appendix 5 Results on effectiveness outcomes.

Multimedia Appendix 6 Detailed health economic results (European Quality of Life 5 Dimensions and the Questionnaire for the Assessment of Medical and Nonmedical Resource Utilization in Mental Disorders).

## Abbreviations

GAD-7: Generalized Anxiety Disorder-7
INEP: Inventory for the Assessment of Negative Effects of Psychotherapy
PACIC: Patient Assessment of Chronic Illness Care
PHQ-9: Patient Health Questionnaire-9
PROVIDE-B: improving cross-sectoral collaboration between primary and psychosocial care: an implementation study on video consultations-B
RAS-G: Recovery Assessment Scale-German
SSD-12: Somatic Symptom Disorder-B Criteria Scale
