# Supplementary material for: Mental Health Specialist Video Consultations Versus Treatment-as-Usual for Patients With Depression or Anxiety Disorders in Primary Care: Randomized Controlled Feasibility Trial
Source: JMIR Ment Health. 2021 Mar 12;8(3):e22569. doi: 10.2196/22569 (PMC7998325; doi:10.2196/22569)
Supplement: Multimedia Appendix 1 [file mental_v8i3e22569_app1.docx]

**APPENDIX 1. Detailed description of the intervention by the MHS**

| **Consultation no.** | **Contents** | |
| --- | --- | --- |
| 1 | **Development of a working alliance, specialized diagnostics** | **Mutual development of a treatment plan** |
| 2 | **Defining/clarifying the central problem, goal setting, and “emotional mindfulness”** |  |
|  | Feedback of specific diagnosis to the general practitioner |  |
| 3 | **Maximized effort to support patient in expressing her/his (avoided) affects.**  At this point, the MHS maximizes her/his effort in supporting patient with experiencing and expressing her/his (avoided) affects. Ideally, the patient’s narrative and the related affects can be linked to the conflictual area and more adaptive responses can be fostered through defense, affect, and/or self-restructuring. Against the background of dysfunctional relationship patterns, the patient and the MHS will generate and negotiate realistic solutions according to the preferences of the patient. At this point the latest, the MHS should ask herself/himself whether the patient will need further treatment, i.e. a referral to social services, specialist care and/or other community resources |  |
| 4 | **Empowering the patient and deciding on referral to specialist treatment.**  The initiation and implementation of the discussed solutions will be evaluated. The MHS empowers the patient to take an active role in the treatment and pays immediate attention to any medical, legal, and/or family crises. While the MHS provides direct support in tackling with the crises pragmatically, she/he adheres to the discussion of relationship conflicts [46]. If the patient can be stabilized, the MHS will empathically address the interactional patterns associated with the crisis. Eventually, the patient and the MHS will decide on any referrals to specialist treatment or community resources. In doing so, the MHS will anticipate potential care gaps and consider the degree of interference with daily activities caused by the patient’s symptoms along with PHQ-9 (1-4 points: no need for action, 5-9 points: monitor for any deterioration, 10 or greater: consider active treatment) and/or GAD-7 scores obtained after the third consultation. During the entire session, the MHS again highlights any potential resources of social support for the patient. If applicable, the MHS also activates health-promoting behavior (sleep hygiene, eating diary, relaxation etc.). |  |
| 5 | **Termination, review, relapse prevention, and, if applicable, outlook to next treatment step/referral.**  Patient and MHS review what they have done, thoroughly discuss steps to prevent relapses and, if applicable, the MHS will provide an outlook to the next treatment step which may or may not include referral to specialist mental health services. |  |
