# Supplementary material for: Mental Health Specialist Video Consultations Versus Treatment-as-Usual for Patients With Depression or Anxiety Disorders in Primary Care: Randomized Controlled Feasibility Trial
Source: JMIR Ment Health. 2021 Mar 12;8(3):e22569. doi: 10.2196/22569 (PMC7998325; doi:10.2196/22569)
Supplement: Multimedia Appendix 2 [file mental_v8i3e22569_app2.docx]

**APPENDIX 2. Detailed description of the dimensions of the Recovery Assessment Scale – German version (RAS-G)**

| **Dimension** | **Description** |
| --- | --- |
| **GSO – Goal and success orientation** | Having a desire to succeed and being able to meet goals |
| **NDS – No domination by symptoms** | Psychiatric symptoms are no longer the center or focus of the person’s life |
| **PCH – Personal confidence and hope** | Statements about respondents liking themselves, having hope for the future, and being able to handle stress |
| **RO – Reliance on others** | Importance of others during course of recovery |
| **WAH – Willingness to ask others for help** | Seeking help from others during course of recovery |

**Reference**

Patrick W. Corrigan, Psy.D., Mark Salzer, Ph.D., Ruth O. Ralph, Ph.D., Yvette Sangster, Lorraine Keck, M.D., Examining the Factor Structure of the Recovery Assessment Scale, Schizophrenia Bulletin, Volume 30, Issue 4, 2004, Pages 1035–1041, https://doi.org/10.1093/oxfordjournals.schbul.a007118
