# Supplementary material for: Mental Health Specialist Video Consultations Versus Treatment-as-Usual for Patients With Depression or Anxiety Disorders in Primary Care: Randomized Controlled Feasibility Trial
Source: JMIR Ment Health. 2021 Mar 12;8(3):e22569. doi: 10.2196/22569 (PMC7998325; doi:10.2196/22569)
Supplement: Multimedia Appendix 5 [file mental_v8i3e22569_app5.docx]

**APPENDIX 5. Results of clinical outcomes**

| **Measure** | **Time Point** | **Intervention** | | **Treatment-as-usual** | | **Total** | | **Effect size rank-biserial *r* [95% CI]^m^** | **P value (Mann-Whitney U-Test)** |
| --- | --- | --- | --- | --- | --- | --- | --- | --- | --- |
|  |  | **N^a^** | **M^b^ (SD)^c^** | **N^a^** | **M^b^ (SD)^c^** | **N^a^** | **M^b^ (SD)^c^** |  |  |
| **PHQ-9^d^** | **Baseline** | 23 | 12.1 (3.40) | 27 | 14.1 (4.27) | 50 | 13.2 (3.99) | 0.27 [0.00 , 0.53] | 0.06 |
|  | **Follow-up** | 22 | 8.7 (4.76) | 23 | 9.8 (6.3) | 45 | 9.3 (5.56) | 0.02 [-0.27 , 0.28] | 0.92 |
|  | **Change from baseline** | 22 | 3.2 (4.03) | 23 | 4.5 (5.50) | 45 | 3.9 (4.83) | -0.15 [-0.41 , 0.11] | 0.29 |
| **GAD-7^e^** | **Baseline** | 23 | 9.9 (4.09) | 27 | 11.7 (4.37) | 50 | 10.9 (4.29) | 0.22 [-0.07 , 0.46] | 0.12 |
|  | **Follow-up** | 22 | 7.9 (4.95) | 23 | 8.6 (5.82) | 45 | 8.2 (5.36) | 0.02 [-0.26 , 0.29] | 0.79 |
|  | **Change from baseline** | 22 | 1.9 (4.42) | 23 | 3.0 (5.41) | 45 | 2.4 (4.93) | -0.13 [-0.4 , 0.16] | 0.37 |
| **SSD-12^f^** | **Baseline** | 23 | 21.6 (8.56) | 27 | 23.4 (12.11) | 50 | 22.6 (10.57) | 0.09 [-0.17 , 0.37] | 0.51 |
|  | **Follow-up** | 22 | 17.0 (9.54) | 23 | 19.5 (12.19) | 45 | 18.3 (10.92) | 0.12 [-0.18 , 0.39] | 0.40 |
|  | **Change from baseline** | 22 | 4.4 (10.80) | 23 | 2.9 (6.88) | 45 | 3.6 (8.94) | 0.07 [-0.21 , 0.34] | 0.61 |
| **RAS-G: GSO^g^** | **Baseline** | 23 | 7.0 (1.92) | 27 | 6.8 (2.42) | 50 | 6.9 (2.18) | 0.01 [-0.27 , 0.28] | 0.93 |
|  | **Follow-up** | 22 | 7.6 (2.17) | 23 | 6.9 (2.29) | 45 | 7.3 (2.24) | 0.16 [-0.13 , 0.41] | 0.27 |
|  | **Change from baseline** | 22 | 0.5 (1.68) | 23 | 0.3 (1.66) | 45 | 0.4 (1.66) | 0.11 [-0.17 , 0.40] | 0.43 |
| **RAS-G: NDS^h^** | **Baseline** | 23 | 4.6 (1.59) | 25 | 4.9 (1.79) | 48 | 4.7 (1.69) | -0.10 [-0.39 , 0.19] | 0.47 |
|  | **Follow-up** | 22 | 6.2 (2.56) | 22 | 5.7 (2.62) | 44 | 5.9 (2.57) | 0.09 [-0.19 , 0.35] | 0.55 |
|  | **Change from baseline** | 22 | 1.8 (2.56) | 20 | 0.9 (2.30) | 42 | 1.3 (2.46) | 0.19 [-0.09 , 046] | 0.18 |
| **RAS-G: PCH^i^** | **Baseline** | 23 | 16.0 (3.61) | 27 | 15.0 (2.72) | 50 | 15.5 (3.16) | 0.22 [-0.06 , 0.51] | 0.12 |
|  | **Follow-up** | 22 | 17.9 (3.24) | 23 | 17.3 (3.57) | 45 | 17.6 (3.39) | 0.06 [-0.25 , 0.33] | 0.66 |
|  | **Change from baseline** | 22 | 1.7 (3.64) | 23 | 2.0 (2.87) | 45 | 1.9 (3.23) | -0.07 [-0.38 , 0.22] | 0.62 |
| **RAS-G: RO^j^** | **Baseline** | 23 | 8.5 (1.70) | 26 | 8.0 (1.31) | 49 | 8.2 (1.51) | 0.18 [-0.14 , 0.46] | 0.22 |
|  | **Follow-up** | 22 | 9.0 (1.48) | 23 | 8.9 (1.52) | 45 | 8.9 (1.48) | 0.04 [-0.26 , 0.33] | 0.79 |
|  | **Change from baseline** | 22 | 0.5 (1.87) | 22 | 0.6 (1.65) | 44 | 0.6 (1.74) | -0.11 [-0.38 , 0.18] | 0.46 |
| **RAS-G: WAH^k^** | **Baseline** | 23 | 11.0 (2.18) | 27 | 10.5 (2.69) | 50 | 10.8 (2.5) | 0.16 [-0.12 , 0.40] | 0.35 |
|  | **Follow-up** | 22 | 12.4 (2.42) | 23 | 11.4 (3.59) | 45 | 11.9 (3.08) | 0.08 [-0.21 , 0.36] | 0.56 |
|  | **Change from baseline** | 22 | 1.4 (2.72) | 23 | 0.5 (2.37) | 45 | 0.9 (2.56) | 0.07 [-0.23 , 0.37] | 0.65 |
| **PACIC–Short Form ^l^** | **Baseline** | 23 | 2.4 (0.75) | 27 | 2.4 (0.87) | 50 | 2.4 (0.81) | 0.04 [-0.25 , 0.31] | 0.81 |
|  | **Follow-up** | 22 | 2.5 (0.93) | 23 | 2.5 (0.74) | 45 | 2.5 (0.83) | 0.02 [-0.25 , 0.29] | 0.91 |
|  | **Change from baseline** | 22 | 0.2 (0.86) | 23 | 0.1 (1.03) | 45 | 0.2 (0.94) | 0.00 [-0.25 , 0.29] | 0.99 |

^a^ N = number of cases; ^b^ M = mean; ^c^ SD = standard deviation; ^d^ PHQ-9 = Patient Health Questionnaire; ^e^ GAD-7 = Generalized Anxiety Disorder 7; ^f^ SSD-12 = Somatic Symptom Disorder-B Criteria Scale; ^g^ RAS-G: GSO = Recovery Assessment Scale (German version): Goal and success orientation; ^h^ RAS-G: NDS = Recovery Assessment Scale (German version): No domination by symptoms; ^i^ RAS-G: PCH = Recovery Assessment Scale (German version): Personal confidence and hope; ^j^ RAS-G: RO = Recovery Assessment Scale (German version): Reliance on others; ^k^ RAS-G: WAH = Recovery Assessment Scale (German version): Willingness to ask others for help; ^l^ PACIC–Short Form = Patient Assessment of Chronic Illness Care–Short Form; ^m^ = confidence intervals by bootstrap with *R* = 1000 replications.
