# Supplementary material for: Mental Health Specialist Video Consultations Versus Treatment-as-Usual for Patients With Depression or Anxiety Disorders in Primary Care: Randomized Controlled Feasibility Trial
Source: JMIR Ment Health. 2021 Mar 12;8(3):e22569. doi: 10.2196/22569 (PMC7998325; doi:10.2196/22569)
Supplement: Multimedia Appendix 6 [file mental_v8i3e22569_app6.docx]

**APPENDIX 6. Detailed health economic results (EQ-5D and the FIMPsy)**

Intervention related costs, health related quality of life, and health care utilization will inform a health economic evaluation of a subsequent effectiveness trial. Regarding health care utilization only resource use is provided. Missing values in the EQ-5D and FIMPsy data were not imputed. In the full health economic evaluation as part of the main trial, missing values will be imputed by multiple imputation.

**Intervention related cost**s

Monthly access costs to the video consultation platform were 9.99€. Four accounts over seven months were used which resulted in 279.72€. Overall, 102 video consultations were conducted. According to prices of 2019, when the trial was started, the salary of a psychotherapist was 89.60€ per video consultation (<https://www.kbv.de/media/sp/EBM_Gesamt___Stand_2._Quartal_2018.pdf>) which resulted in 9,139.20€ for psychotherapist salaries. Nine supervisions with five psychotherapists each took place and, thus, yielded costs of 4,032.00€. In total, intervention related costs summed up to 13,450.92€ for 23 patients.

**Health related quality of life (EQ-5D-5L)**

The feasibility trial was also used to investigate outcomes that will be employed in a health economic evaluation of a subsequent effectiveness trial. The mean EQ-5D-5L value tended to increase in the intervention and the control group (see Table A2.1.).

In the main trial, a cost-utility analysis and a cost-effectiveness analysis will be performed. Regarding the cost-utility analysis, quality adjusted life years (QALYs) will serve as outcomes based on the EQ-5D-5L quality of life measure which is evaluated by a German tariff.^[[1]](#footnote-1)^

Table A2.1. EQ-5D-5L values and VAS-values for intervention and control group

|  | **Intervention group** | | | **Control group** | | |  |
| --- | --- | --- | --- | --- | --- | --- | --- |
|  | **Mean** | **SD** | **N** | **Mean** | **SD** | **N** | ***p*-value^a^** |
| **EQ-5D-5L value Baseline** | 0.753 | 0.141 | 23 | 0.652 | 0.258 | 27 | 0.243 |
| **EQ-5D-5L value Follow-Up** | 0.876 | 0.132 | 22 | 0.803 | 0.237 | 23 | 0.445 |
| **Diff. EQ-5D-5L values** | 0.115 | 0.119 | 22 | 0.143 | 0.317 | 23 | 0.838 |
| **VAS Baseline** | 61.739 | 17.163 | 23 | 55.615 | 15.344 | 26 | 0.075 |
| **VAS Follow-Up** | 70.409 | 20.572 | 22 | 68.652 | 21.073 | 23 | 0.621 |
| **Diff. VAS** | 8.364 | 21.086 | 22 | 14.682 | 21.753 | 22 | 0.444 |

^a^Mann-Whitney U-Test; EQ-5D-5L = European Quality of Life 5 Dimensions; VAS = Visual Analogue Scale

**Health care utilization**

Health care utilization was recorded at baseline and at the follow-up assessment using the questionnaire for the Assessment of Medical and non-Medical Resource Utilization in Mental Disorders (FIMPsy).^[[2]](#footnote-2)^ In particular, the questionnaire comprises information regarding hospital stays, psychiatric emergencies, psychiatric outpatient clinic contacts, physician contacts, medication intake of psychiatric medication, counselling and assistance, employment, and absenteeism.

At baseline and at the follow-up assessment, hospital stays and psychiatric emergencies are rare events in the intervention and the control group (see table A2.2.). The number of psychiatric outpatient clinic contacts seem to be larger at the follow-up, especially for the intervention group. Yet, only three individuals of the IG induced the 21 contacts. The seven contacts dedicated to the control group were induced by two individuals. Individuals of the intervention and the control group contacted rather general practitioners than physicians of other professions. The sum of provided psychotherapy by psychotherapists, specialists in psychosomatic medicine, and psychiatrists is larger in the CG (Baseline: 29; Follow-Up: 61) than in the intervention group (Baseline: 7; Follow-Up: 38) which is again driven by few individuals (individuals of the CG with at least one psychotherapy contact at baseline: 6, at follow-up: 12; individuals of the IG with at least one psychotherapy contact at baseline: 6, at follow-up: 8).

Medication intake was recorded for psychiatric medication (see table A2.3.). Most individuals consumed not any or one drug. Thus, the total amount of consumed psychiatric medication is similar to the number of individuals who consumed at least one drug. While five individuals and 13 individuals of the intervention group and control group, respectively, consumed at least one psychiatric drug at baseline, at follow-up seven individuals of the intervention group and 12 individuals of the control group took at least one psychiatric drug.

Regarding use of counselling and assistance (see table A2.4), few individuals out of the intervention and control group made use of possible options, e.g. the helpdesk for persons with psychological problems and self-help groups.

From employment and absenteeism data one can infer indirect costs in form of productivity losses. The total number of individuals employed did not change much during the intervention for the intervention and control group (see table A2.5). The total number of absenteeism days in the past 30 days increases for the intervention group while it decreases for the control group. This might be driven by lesser people employed and missing values in the intervention and control group.

Table A2.2. Inpatient and outpatient health care utilization

|  | **Intervention group** | | | **Control Group** | | | **Overall** | | |
| --- | --- | --- | --- | --- | --- | --- | --- | --- | --- |
|  | **Number of** | **N** | **Missings** | **Number of** | **N** | **Missings** | **Number of** | **N** | **Missings** |
| **Number of Hospital stays** |  |  |  |  |  |  |  |  |  |
| Baseline | 0 | 23 | 0 | 0 | 26 | 1 | 0 | 49 | 1 |
| Follow-Up | 1 | 22 | 1 | 1 | 23 | 4 | 2 | 45 | 5 |
| **Number of psychiatric outpatient clinic contacts** |  |  |  |  |  |  |  |  |  |
| Baseline | 4 | 22 | 1 | 4 | 27 | 0 | 8 | 49 | 1 |
| Follow-Up | 21 | 22 | 1 | 7 | 23 | 4 | 28 | 45 | 5 |
| **Number of psychiatric emergency contacts** |  |  |  |  |  |  |  |  |  |
| Baseline | 0 | 23 | 0 | 0 | 27 | 0 | 0 | 50 | 0 |
| Follow-Up | 1 | 22 | 1 | 0 | 23 | 4 | 1 | 45 | 5 |
| **Number of physician contacts** |  |  |  |  |  |  |  |  |  |
| General practitioner baseline | 92 | 23 | 0 | 146 | 27 | 0 | 238 | 50 | 0 |
| General practitioner follow-Up | 111 | 22 | 1 | 115 | 23 | 4 | 236 | 45 | 5 |
| Internist baseline | 5 | 23 | 0 | 5 | 27 | 0 | 10 | 50 | 0 |
| Internist follow-Up | 3 | 22 | 1 | 4 | 23 | 4 | 7 | 45 | 5 |
| Cardiologist baseline | 2 | 23 | 0 | 4 | 27 | 0 | 6 | 50 | 0 |
| Cardiologist follow-Up | 0 | 22 | 1 | 4 | 23 | 4 | 4 | 45 | 5 |
| Orthopedist baseline | 4 | 23 | 0 | 3 | 27 | 0 | 7 | 50 | 0 |
| Orthopedist follow-Up | 5 | 22 | 1 | 3 | 23 | 4 | 8 | 45 | 5 |
| Dermatologist baseline | 5 | 23 | 0 | 3 | 27 | 0 | 8 | 50 | 0 |
| Dermatologist follow-Up | 3 | 22 | 1 | 6 | 23 | 4 | 9 | 45 | 5 |
| Neurologist baseline | 3 | 23 | 0 | 4 | 27 | 0 | 7 | 50 | 0 |
| Neurologist follow-Up | 2 | 22 | 1 | 2 | 23 | 4 | 4 | 45 | 5 |
| Psychotherapist baseline | 4 | 23 | 0 | 12 | 27 | 0 | 16 | 50 | 0 |
| Psychotherapist follow-Up | 37 | 22 | 1 | 38 | 23 | 4 | 75 | 45 | 5 |
| Specialist in psychosomatic medicine baseline | 1 | 23 | 0 | 6 | 27 | 0 | 7 | 50 | 0 |
| Specialist in psychosomatic medicine follow-Up | 0 | 22 | 1 | 0 | 23 | 4 | 0 | 45 | 5 |
| Psychiatrist baseline | 2 | 23 | 0 | 11 | 27 | 0 | 13 | 50 | 0 |
| Psychiatrist follow-Up | 1 | 22 | 1 | 23 | 23 | 4 | 24 | 45 | 5 |
| Other practitioner baseline | 8 | 23 | 0 | 7 | 27 | 0 | 15 | 50 | 0 |
| Other practitioner follow-Up | 15 | 22 | 1 | 6 | 23 | 4 | 21 | 45 | 5 |

Table A2.3. Medication intake of psychiatric medication

|  | **IG** | | | **CG** | | | **Overall** | | |
| --- | --- | --- | --- | --- | --- | --- | --- | --- | --- |
|  | **Number of** | **N** | **Missings** | **Number of** | **N** | **Missings** | **Number of** | **N** | **Missings** |
| **Number of psychiatric medications** |  |  |  |  |  |  |  |  |  |
| Baseline | 7 | 23 | 0 | 17 | 27 | 0 | 24 | 50 | 0 |
| Follow-Up | 7 | 22 | 1 | 19 | 23 | 4 | 26 | 45 | 5 |

Table A2.4. Counselling and assistance

|  | **IG** | | | **CG** | | | **Overall** | | |
| --- | --- | --- | --- | --- | --- | --- | --- | --- | --- |
|  | **Number of** | **N** | **Missings** | **Number of** | **N** | **Missings** | **Number of** | **N** | **Missings** |
| **Helpdesk for persons with psychological problems** |  |  |  |  |  |  |  |  |  |
| Baseline | 1 | 23 | 0 | 1 | 26 | 1 | 2 | 49 | 1 |
| Follow-Up | 2 | 22 | 1 | 1 | 23 | 4 | 3 | 45 | 5 |
| **Socio-psychiatric services** |  |  |  |  |  |  |  |  |  |
| Baseline | 0 | 23 | 0 | 0 | 26 | 1 | 0 | 49 | 1 |
| Follow-Up | 0 | 22 | 1 | 0 | 23 | 4 | 0 | 45 | 5 |
| **Daycare center for persons with psychological problems** |  |  |  |  |  |  |  |  |  |
| Baseline | 0 | 23 | 0 | 0 | 26 | 1 | 0 | 49 | 1 |
| Follow-Up | 0 | 22 | 1 | 0 | 23 | 4 | 0 | 45 | 5 |
| **Self-help group for persons with psychological problems** |  |  |  |  |  |  |  |  |  |
| Baseline | 0 | 23 | 0 | 1 | 26 | 1 | 1 | 49 | 1 |
| Follow-Up | 0 | 22 | 1 | 2 | 23 | 4 | 2 | 45 | 5 |
| **Other counseling and assistance** |  |  |  |  |  |  |  |  |  |
| Baseline | 3 | 23 | 0 | 1 | 26 | 1 | 4 | 49 | 1 |
| Follow-Up | 0 | 22 | 1 | 0 | 23 | 4 | 0 | 45 | 5 |

Table A2.5. Employment and absenteeism

|  | **IG** | | | **CG** | | | **Overall** | | |
| --- | --- | --- | --- | --- | --- | --- | --- | --- | --- |
|  | **Number of** | **N** | **Missings** | **Number of** | **N** | **Missings** | **Number of** | **N** | **Missings** |
| **Number of employed individuals** |  |  |  |  |  |  |  |  |  |
| Baseline | 17 | 22 | 1 | 17 | 27 | 0 | 34 | 49 | 1 |
| Follow-Up | 16 | 22 | 1 | 13 | 23 | 4 | 29 | 45 | 5 |
| **Number of total weekly working hours** |  |  |  |  |  |  |  |  |  |
| Baseline | 588 | 22 | 1 | 462 | 27 | 0 | 1050 | 49 | 1 |
| Follow-Up | 557 | 22 | 1 | 330 | 23 | 4 | 887 | 45 | 5 |
| **Number of individuals not able to do their daily work on at least one day in the past 30 days** |  |  |  |  |  |  |  |  |  |
| Baseline | 13 | 22 | 1 | 14 | 23 | 4 | 36 | 45 | 5 |
| Follow-Up | 10 | 20 | 3 | 7 | 20 | 7 | 17 | 40 | 10 |
| **Number of total absenteeism days in the past 30 days** |  |  |  |  |  |  |  |  |  |
| Baseline | 139 | 22 | 1 | 175 | 23 | 4 | 314 | 45 | 5 |
| Follow-Up | 208 | 20 | 3 | 127 | 20 | 7 | 335 | 40 | 10 |

1. Ludwig, K., Graf von der Schulenburg, J.-M., Greiner, W.: German value set for the EQ-5D-5L. Pharmacoeconomics (2018). https ://doi.org/10.1007/s4027 3-018-0615-8. [↑](#footnote-ref-1)
2. Grupp H, König H-H, Riedel-Heller S, Konnopka A. FIMPsy–Fragebogen zur Inanspruchnahme medizinischer und nicht medizinischer Versorgungsleistungen bei psychischen Erkrankungen: Entwicklung und Verwendung. Psychiatrische Praxis 2018;45(02):87–94. [↑](#footnote-ref-2)
